# Enemy-Risk Effects in Parasitoid-Exposed Diamondback Moth Larvae: Potential Mediation of the Interaction by Host Plants

**DOI:** 10.3390/insects13090818

**Published:** 2022-09-07

**Authors:** Naoki Kihata, Ikkei Shikano

**Affiliations:** 1 Colby College, Mayflower Hill Drive, Waterville, ME 04901, USA; 2 Department of Plant and Environmental Protection Sciences, College of Tropical Agriculture and Human Resources, University of Hawai’i at Mānoa, 3050 Maile Way, Gilmore Hall 513, Honolulu, HI 96822, USA

**Keywords:** fear, insect herbivore, multitrophic interaction, non-consumptive effect, predator-prey interaction, tritrophic interaction

## Abstract

**Simple Summary:**

While consumption by predators directly reduces prey populations, some studies have shown that even the mere threat of predation can negatively impact the health and fitness of prey. This is referred to as the enemy-risk effect. We investigated enemy-risk effects in caterpillars (larval stage of the diamondback moth, *Plutella xylostella*) feeding on two different host plants in the presence and absence of the parasitoid wasp, *Diadegma insulare*. We found that the threat of attack can slow down caterpillar development and produce smaller adult moths. These physiological changes coincided with a change in caterpillar behavior to feed on older foliage, and the host plant species appeared to influence the severity of the enemy-risk effects.

**Abstract:**

Enemy-risk effects (i.e., non-consumptive effects) describe the non-lethal fitness costs incurred by animals when they perceive a risk of predation. These effects can result from fear-associated changes in behavior and physiology. Diamondback moth larvae (*Plutella xylostella*) are known to violently wriggle backwards and drop from their host plants, usually suspending themselves with a silk thread, when threatened by predators and parasitoids. Here, we investigated the developmental costs associated with this behavior when larvae were exposed to its specialist parasitoid wasp (*Diadegma insulare*). Additionally, the structural and chemical properties of plants are well-known to influence predation and parasitism rates of herbivorous insects. Yet, few studies have examined the influence of plants on enemy-risk effects. Therefore, we examined the developmental costs associated with parasitism risk on two host plants. Diamondback moth larvae were placed on either cabbage or Virginia pepperweed plants and exposed to gravid parasitoids with truncated ovipositors, which prevented piercing of the host cuticle without affecting host searching and attacking behaviors. On Virginia pepperweed, risk of parasitism resulted in reduced larval weight gain, longer development time, and smaller adult size compared to larvae that were not exposed to parasitoids. However, on cabbage, parasitoid exposure prolonged development time but had no significant effects on larval weight gain and adult size. On both plants, parasitoid-exposed larvae were found feeding on older foliage than younger foliage. Our findings demonstrate that the enemy-escape behavior of diamondback moths has developmental costs and that plants may mediate the intensity of these enemy-risk effects.

## 1. Introduction

Deciphering species interactions through their predator–prey relationships and the transfer of energy through trophic levels is pivotal to understanding the functioning of ecosystems. These trophic dynamics can be generalized into two categories: consumptive and non-consumptive effects. Consumptive effects describe prey mortality from predation, while enemy-risk or non-consumptive effects describe a reduction in prey fitness associated with the costs of risk-induced behavioral changes [1,2,3]. For example, pea aphids drop from plants to escape predators such as ladybugs. This escape mechanism can reduce aphid fitness by wasting feeding time [4]. It can also expose aphids to predators and pathogens on the ground [5]. Though enemy-risk effects can be as important to the fluctuation of prey populations as consumptive effects, a knowledge gap remains when limiting enemy-risk effects to the interaction between two species [6].

Plants can play an important role in the severity of enemy-risk effects [2]. For instance, grasshoppers, which mostly feed on grass in the absence of predators, fed on less nutritious forbs when they were exposed to spiders. Forbs have more complex structures, which was suggested to provide better hiding places for the grasshoppers [7,8]. While generalist herbivores can change their food plants in the presence of predators, specialist insects have fewer options and will resort to eating different parts of a plant or reducing foraging activity. Thus, for specialist herbivores and generalists with limited movement between plants, the nutritional heterogeneity within a plant and the structure of the plant may have strong influences on enemy-risk effects. In larvae of the European grapevine moth, *Lobesia botrana*, threat of parasitism resulted in accelerated development to pupation [9]. This could be an evolved strategy to shorten the susceptible period of *L. botrana* (larval stage) to the parasitoid through a re-allocation of resources from growth to reproduction [10]. However, the study restricted the *L. botrana* larvae to a nutritionally homogenous semi-artificial diet. If enemy risk can induce behavioral changes that alter host plant choice and/or feeding location within a plant and hence nutritional quality for the insect, insect developmental responses to enemy risk may differ from responses observed on nutritionally fixed diets.

The diamondback moth (DBM) is a specialist of plants in the Brassicaceae family. When a DBM larva is attacked by parasitoids and predators, it will violently wriggle backwards and drop from the host plant, usually suspending itself with a silk thread [11,12]. This dropping and hanging in response to attacks is a widespread behavior among larvae in the order Lepidoptera [13,14]. While this escape behavior can be lifesaving, there could be fitness costs associated with the behavior. Therefore, we measured fitness costs associated with enemy avoidance behavior in DBM. We compared several developmental measures in DBM (larval weight, development time, pupa length, adult longevity, and forewing length) when they were exposed or not exposed to parasitoids during the larval stage. The parasitoids were prevented from injuring and ovipositing in the DBM larvae by surgically truncating and blunting their ovipositor. Thus, DBM larvae could perceive the enemy risk via visual, olfactory, and tactile cues.

We used the specialist parasitoid of DBM, *Diadegma insulare*, and two host plants, cabbage (*Brassica oleracea* var. *capitata*) and Virginia pepperweed (VPW; *Lepidium virginicum*). The two plants were selected to try to capture some of the wide variation in DBM host plants. First, cabbage and VPW differ in host plant quality. DBM have a strong oviposition preference for VPW over cabbage [15], and when larvae completed development on the two plants, adult longevity and flight duration capacity were greater on VPW than on cabbage when adjusted for adult size [16]. Second, the plants differ structurally. Cabbage leaf age changes radially outward, such that nutritional quality and the concentrations of allelochemicals, which changes with leaf age, varies horizontally within a plant. DBM larvae prefer to feed on younger leaves of *Brassica oleracea*, as they are better quality food for larval growth than mature and senescent leaves [17]. In contrast, VPW bolts when they are about 9 weeks old, as used in the present study. This results in a vertical gradient of foliage age, with most of the newer growth at the top of the bolting plant and older, denser vegetation close to the soil surface. From our observations, DBM larvae prefer to feed on the newer growth of VPW. Therefore, we also assessed whether the dropping behavior associated with parasitoid avoidance results in DBM larvae feeding on the lower, older vegetation of VPW.

## 2. Materials and Methods

### 2.1. Plants and Insects

Plants were grown in an air-conditioned roof-top (7th floor) greenhouse at Gilmore Hall, University of Hawai’i at Mānoa, which was maintained at approximately 22–26 °C. Plants consisted of individual 6-week-old cabbage plants (*B. oleracea* var. K-K Cross; Holmes Seed Company, Canton, OH, USA) or clusters of four 9-week-old VPW plants, which approximately matched the biomass of the two plant species, grown in 15 × 14 cm (D × H) round plastic pots. VPW seeds were collected from naturally growing plants on the University of Hawai’i at Mānoa campus. Plants were grown in professional growing mix (Sunshine Mix #4; Sun Gro Horticulture, Agawam, MA, USA) and fertilized directly at sowing with Osmocote Plus slow-release indoor and outdoor plant food (15-9-12, N-P-K; The Scotts Miracle-Gro Company LLC, Marysville, OH, USA). All plants were grown in insect-proof tents (BugDorm-2400F; Mega View Science Co., Ltd., Taichung City, Taiwan) until use in the experiment.

DBM larvae were collected from a commercial kale farm in Kekaha, Kauai, on 20 April 2021 and maintained in the lab on cabbage. Parasitoid (*D. insulare*) cocoons were collected from a commercial cabbage farm in Kula, Maui, on 2 June 2021. Only two species of DBM parasitoids are known to occur in Kula (*Cotesia vestalis* and *D. insulare*), and the two are easily distinguished by the appearance of their cocoons. *D. insulare* prefers to oviposit in 2nd to 4th instar larvae [11,12]. Adults that emerged from the collected cocoons were provided with 2nd and 3rd instar DBM larvae in the lab for oviposition, and the resulting offspring wasps were used in the experiment.

To induce non-consumptive effects in DBM larvae, the ovipositor of each parasitoid was excised to less than 50% of its length, which also blunted the ovipositor to prevent penetration of the DBM cuticle. A gravid parasitoid, which had not been exposed to plants and hosts since emergence, was temporarily immobilized by placing in ice water for 1 min and the ovipositor was cut perpendicularly with micro-scissors. The wasps were used for the experiment 24 h later. Preliminary surgeries and observations indicated that the host searching behavior and oviposition attempts were not noticeably affected. Additionally, no parasitoid larvae emerged from parasitoid-exposed DBM larvae in our study, indicating that the surgery successfully prevented oviposition. The intended effect of our surgical treatment was similar to studies that glued the chelicera of spiders to induce enemy-risk effects without physically harming the prey [7,8].

### 2.2. Experimental Design

For experimental setup, a 13 × 40 cm (D × H) clear plexiglass tube enclosure with fine mesh covering the top and six 8 cm diameter windows on the sides for ventilation was placed over the cabbage or VPW cluster to fully enclose the plants in each pot. Plants were left for 24 h to acclimate to the new conditions. Groups of 15, 2nd instar DBM larvae (<12 h since molt), which were reared on their respective food plant species, were placed into each plant enclosure. Half the enclosures for cabbage and VPW received two female parasitoids with truncated ovipositors. One water-soaked and one honey-soaked cotton ball was placed into each parasitoid-containing enclosure. All enclosures were placed next to a window in a temperature-controlled room (25 °C). In total, six enclosures were set up for each plant by parasitoid treatment (2 × 2 factorial design); two enclosures of each treatment were set up on 10 July 2021, one each on 12 July 2021, and three each on 29 July 2021. After 72 h, the enclosures were opened, and parasitoids were confirmed to be alive. One enclosure (VPW; 29 July 2021) had dead parasitoids and was excluded from analyses. Location of DBM larvae were assessed from five VPW in each treatment. Location of larvae on cabbage were only assessed from the three plants on 29 July 2021. For cabbage, the location of larvae was leaf position; for VPW, it was the top 10 cm of the bolting new growth or in the denser foliage below. All DBM larvae were collected and weighed (nearest 0.01 mg) and placed in individual 30 mL plastic cups and provided with the foliage it was found on and additional foliage from a similar position on the plant. The DBM sex, days to pupation, pupa length, adult longevity (without food or water), and adult forewing length were measured.

### 2.3. Statistical Analyses

Larval weight, development time, pupa length, adult longevity, and forewing length were analyzed using a mixed model with parasitoid and plant treatments and their interaction included as factors, plant enclosure included as a random effect to account for measurements from 15 larvae in each enclosure, and sex included as a covariate. Larval weight was log10 transformed, and forewing length was square root transformed to meet the assumptions of normality. The location of DBM larvae was analyzed by analysis of variance. All analyses were performed on JMP Pro 16 (SAS Institute Inc., Cary, NC, USA).

## 3. Results

**Figure 1:**
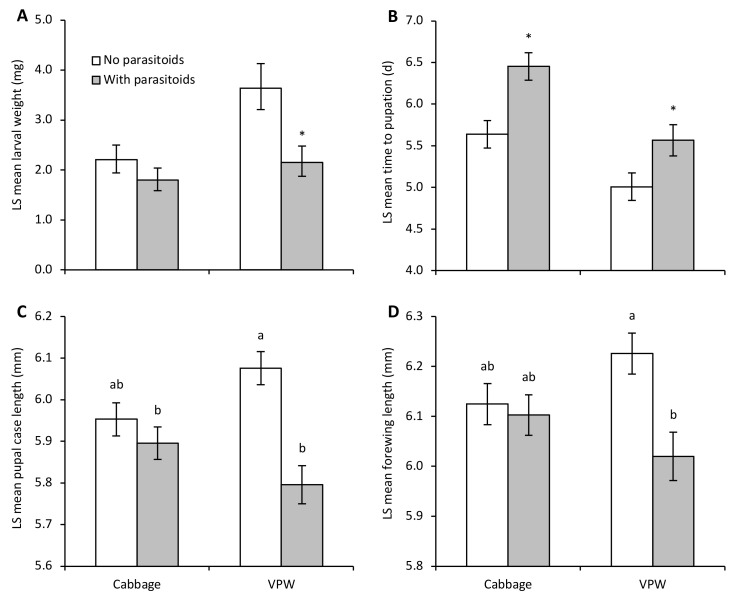
Developmental measures (LS mean ± SE) of diamondback moth on Virginia pepperweed (VPW) and cabbage in enclosures with or without parasitoids. Figure panels present (**A**) larval weight immediately after parasitoid exposure, (**B**) time to pupation, (**C**) pupal case length, and (**D**) forewing length. Different letters above bars indicate significant differences between treatments based on a significant interaction between parasitoid and plant treatments. Asterisks above bars represent significant differences from means contrasts (*p* < 0.05) between parasitoid and no parasitoid enclosures within a plant treatment.

An enemy-risk effect was evident as parasitoid-exposed DBM larvae weighed significantly less than control larvae after the 72 h exposure period even though they could not be physically injured by the parasitoids (parasitoid: F_1,20.0_ = 7.62, *p* = 0.01; plant: F_1,20.1_ = 6.84, *p* = 0.02; parasitoid by plant: F_1,19.1_ = 1.68, *p* = 0.21) (Figure 1A). Larvae on VPW were significantly heavier than those on cabbage plants. While there was no significant interaction between plant and parasitoid-exposure treatments, a means contrast comparing parasitoid-mediated effects separately on each host plant indicated that the presence of parasitoids significantly reduced larval weight on VPW (F_1_ = 8.25, *p* = 0.01) and not on cabbage (F_1_ = 1.30, *p* = 0.27).

Parasitoid-exposed larvae on both plant species took longer to reach the pupal stage than control larvae (parasitoid: F_1,20.5_ = 16.98, *p* = 0.0005); larvae on cabbage took longer to develop than on VPW (plant: F_1,20.9_ = 19.71, *p* = 0.0002; parasitoid by plant: F_1,19.6_ = 0.57, *p* = 0.46) (Figure 1B). Statistically significant enemy-risk effects were only detected in adulthood on VPW, with smaller pupal size (parasitoid by plant: F_1,17.4_ = 7.64, *p* = 0.01) (Figure 1C) and shorter forewings (parasitoid by plant: F_1,19.1_ = 4.59, *p* < 0.05) (Figure 1D). Adults survived longer if they developed on VPW than cabbage (F_1,21.1_ = 8.12, *p* = 0.01), but longevity was not affected by parasitoid exposure (F_1,20.5_ = 1.69, *p* = 0.21; parasitoid by plant: F_1,19.5_ = 2.22, *p* = 0.15). The random factor (i.e., individual host plant enclosures) significantly affected larval weight (Wald *p*-value = 0.004; percentage of total = 48.93), development time (Wald *p*-value = 0.02; percentage of total = 20.13), and adult longevity (Wald *p*-value < 0.05; percentage of total = 11.06).

**Figure 2:**
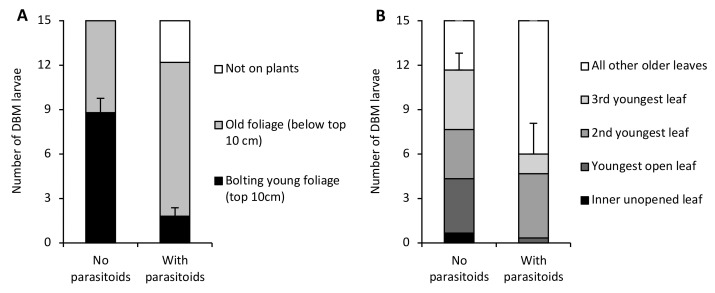
Differences in the location of diamondback moth larvae inside (**A**) Virginia pepperweed (VPW) and (**B**) cabbage enclosures in the absence and presence of parasitoids. Standard error bars are for mean numbers of larvae found on the bolting young foliage of Virginia pepperweed and the mean of the sum of larvae on the innermost unopened and three youngest opened leaves of cabbage.

Without parasitoids, most DBM larvae were found feeding on the new bolting growth in the upper 10 cm of the VPW. However, when parasitoids were present, almost all larvae were found in the older vegetation close to the soil surface (F_1,8_ = 38.28, *p* = 0.0003) (Figure 2A). Additionally, some DBM were found on the wall of the VPW enclosures when parasitoids were present (2.8 ± 1.2 larvae), whereas all DBM were found on the plants when parasitoids were absent (F_1,8_ = 5.44, *p* < 0.05). On cabbage, the average number of larvae on the youngest leaves (innermost unopened and three youngest opened leaves) from each plant tended to be lower in the presence of parasitoids (6.0 ± 2.1 larvae) than in their absence (11.7 ± 1.2 larvae) though not significantly (F_1,4_ = 5.56, *p* = 0.08) (Figure 2B). No larvae were found on the wall of the cabbage enclosures.

## 4. Discussion

Our main finding was that DBM larvae suffered reductions in performance measures when posed with the risk of parasitoid attacks. DBM larvae grew slower in the presence of parasitoids. Our observations indicated that consumption of poorer-quality foliage (i.e., older foliage) in the presence of parasitoids likely contributed to the slower growth rate. Another possible mechanism for the observed enemy-risk effect is due to a reallocation of resources from growth to immune defense. Some insects that detect the presence of parasites or predators have been shown to increase their investment in defenses [18]. In some cases, insects that perceive a risk can prophylactically modulate immune defenses even in the absence of the parasites, such as high conspecific population densities, which increase the probability of disease transmission [19,20]. Since consuming poorer-quality foods usually reduces both growth rate and immune functioning in insects [21], the combination of poorer food quality and reallocation of those limited resources to immune functioning would further impact growth rate. The reduced growth rate and prolonged development of parasitoid-exposed DBM larvae that were maintained on plants in our study was in direct opposition to the accelerated development found in parasitoid-exposed European grapevine moth, *L. botrana*, larvae that were fed on a nutritionally fixed semi-artificial diet [9]. Though these are different insect–parasite–food systems, the contradicting results suggest that diet could be an important factor that influences enemy-risk effects. Host plants and plant parts vary in nutritional quality and can mediate the outcomes of interactions between herbivorous insects with predators, parasites, and pathogens through direct phytochemical toxicity to the natural enemies and altered host immune functioning [21]. Host plants have even been demonstrated to influence the risk-induced prophylactic immune responses of an herbivorous insect [22]. Another important difference between our DBM study and the *L. botrana* study is that the parasitoids were able to physically contact the DBM larvae, while the parasitoids in the *L. botrana* study were not. Therefore, the parasitoid cues perceived by the insect may also play an important role in their physiological and behavioral responses.

We found some differences in performance measures between larvae reared on cabbage and VPW, but there are several limitations when interpreting these plant-mediated effects. First, we found a clear direct effect of parasitoid exposure, represented by reduced larval weight, on VPW but not on cabbage. Second, we found that DBM larvae on VPW were significantly more likely to be hiding and/or feeding in older foliage in the presence of parasitoids than in their absence. Changes in feeding location on cabbage plants were less clear though parasitoid-exposed DBM tended to avoid the youngest foliage. These results agree with previous studies that showed herbivores reduced feeding rate and/or fed on lower-quality resources in the presence of natural enemies [23,24]. While we examined 15 larvae per enclosure, higher numbers of cabbage and VPW enclosures were needed to confidently interpret plant-mediated effects. Unfortunately, we were unable to set up additional replicates, as the *D. insulare* population became completely male biased, which occurs when these parasitoids are laboratory-reared due to frequent haploid parthenogenesis [11], and we were unable to collect more, as they became seasonally unavailable in the field. More detailed studies combined with assessments of nutritional differences of young and old foliage are needed. Third, we found that parasitoid-exposed larvae on VPW yielded smaller pupae and adults with shorter forewings, indicating a long-term enemy-risk effect. Smaller adult size is associated with reduced fecundity in Lepidoptera [25]. Moreover, reduced adult size resulting from poor nutrient intake can induce transgenerational effects that affect egg size, offspring growth rate, and immune functioning [26,27]. An important limitation of our experimental design was that when we recovered larvae after the 72 h parasitoid exposure period, we maintained the larvae on the leaves they were found on (and similar age leaves) until pupation. However, caterpillars are known to actively regulate their nutrient intake [28]. Therefore, restricting their diet to the age of leaves on which they were found limited their ability to acquire an optimal ratio of nutrients. While we did not observe much frass or feeding damage on the younger foliage of VPW in parasitoid-containing enclosures, it is possible that larvae could have moved to those younger parts of the plants when they perceived a lower risk (e.g., during times of the day when parasitoids were less active). Lastly, only parasitoid-exposed DBM larvae in the VPW enclosures were found on the enclosure wall, while all of the larvae in the cabbage enclosures were found on the cabbage plants. This suggests that the avoidance or escape behaviors of DBM were stronger on VPW. However, the intensity of harassment by the parasitoids may have been unrealistic. Fifteen DBM larvae were exposed to two gravid parasitoids for 72 h. We did not observe how many oviposition attempts were made by the parasitoids during that period, but it may have been much higher than what would be encountered by non-parasitized DBM larvae in the field. Thus, further studies on the intensity of host-searching behavior by parasitoids on enemy-risk effects are needed. This should also be performed in the context of other natural enemies because falling to the ground after one oviposition attempt by a parasitoid could be lethal to the DBM if there are pathogens or predators lurking on the soil surface.

Generally, enemy-risk effects allow for three distinctive shifts in an organism’s homeostasis: behavioral, physiological, and morphological [5]. These non-consumptive effects can be induced by tactile, auditory, hormonal, and visual cues of the natural enemies. Behavioral shifts from enemy-risk are the most documented, as these changes happen rapidly and are often reversible. When physically contacted or in adjacent proximity with a natural enemy, DBM larvae wriggle away and either suspend themselves with a silk thread or completely drop off of the plant [11,12]. DBM larvae will also avoid detection by natural enemies by moving away from damaged plant parts after short feeding bouts [12]. In some systems, the behavioral responses of the prey/host have been shown to depend on the attacking natural enemy. When the pea aphid, *Acyrthosiphon pisum,* and the green peach aphid, *Myzus persicae*, each encountered two different parasitoid species, *A. pisum* engaged in the same defensive response of falling off of its host plant, whereas *M. persicae* engaged in different responses [29]. When the parasitoid wasp *Aphidius colemani* was present, *M. persicae* engaged in defensive behaviors, such as kicking and emitting secretions, but when *A. ervi* was present, *M. persicae* did not engage in defensive behaviors and instead spent more time off of the plant [29]. Moreover, even the behaviors of an herbivore’s natural enemies can be influenced by their own natural enemies. Höller et al. [30] found that 92% of adults of the aphid parasitoid, *Aphidius uzbekistanicus,* left an area when they detected chemical volatiles of the adult hyperparasitoid, *Alloxysta victrix*. The chemicals being emitted by *A. victrix* acts as a spacing and sex phermone, which signals *A. uzbekistanicus* adults that their offspring would not survive in the same proximity as the hyperparasitoid [30].

Behavioral shifts originating from the enemy-risk effect often cause physiological shifts, which result in a change in prey development and growth [5]. Predation risk from phytohormonal cues of the stink bug *P. maculiventris* reduced food intake by the generalist herbivore *Trichoplusia ni*, which resulted in a decrease in *T. ni* larval growth rate [31]. However, the enemy-risk effects were attenuated in the specialist herbivore *Manduca sexta*, as it compensated for predator-induced effects by increasing the efficiency of conversion of food energy to longer-term energy resources such as lipids [31]. When *M. sexta* larvae were exposed to the predatory spined soldier bug, *Podisus maculiventris*, *M. sexta* decreased foraging by 32%, but this coincided with a 19% increase in assimilation efficiency and a 17% increase in resting metabolic rate compared to unexposed *M. sexta* [32]. Interestingly, there was a plant-mediated effect in that enemy risk only increased assimilation efficiency and resting metabolic rate in *M. sexta* that foraged on plants with low resistance to feeding (i.e., high-quality host plants) and not on resistant plants (i.e., low-quality host plants) [32]. DBM is also characterized as a specialist herbivore on plants from the *Brassicaceae* family. Our results suggest that enemy-risk effects were more severe on VPW than cabbage. Whether this plant-mediated difference is attributable to changes in metabolism requires further investigation.

In some systems, enemy risk can lead to the manifestation of permanent morphological changes. These morphological changes have been most studied in aquatic arthropods such as *Daphnia pulex*. This organism exhibits dynamic morphological defenses that change in response to predation pressure. For example, long-term exposure to predators, such as the tadpole shrimp, *T. cancriformis*, through direct contact and chemical cues induced increased body volume and tail spine length in *D. pulex* [33]. Some evidence suggests that enemy risk can induce morphological changes in terrestrial arthropods as well. Recently, olfactory cues from the predatory zigzag ladybird beetle, *Cheilomenes sexmaculata*, were shown to induce an increase in the numbers of winged progeny morphs in the cotton aphid, *Aphis gossypii* [34]. Similar morphological effects in progeny have been found when aphids are exposed to parasitoids [35]. We did not observe any noticeable changes in morphology other than shorter forewing lengths in parasitoid-exposed DBM on VPW, which coincided with their smaller pupal size. DBM with longer forewings relative to body size have significantly greater dispersal rates and flight activity [36]. Generally, in insects, longer wings are correlated with lower fecundity, likely due to a trade-off between investment in flight capacity and other life history traits [37]. Thus, the population dynamics of the host/prey and consequently their natural enemies is likely to be modulated by enemy-risk effects that carry over from the larval stage to affect adult dispersal and fecundity.

The impact of plants on enemy-risk effects in our study may be attributable to plant-mediated effects on parasitoid behaviors. Plant complexity, which includes variations in size, heterogeneity, and connectivity, affect the range of host searching areas and overall host-finding success of parasitoids. For example, host-finding success of the parasitoid *Trichogramma evanescens* was higher on artificial plants with a simple structure and low on plants with a complex structure. This variability in parasitism rate was most closely associated with connectivity of the plants, decreasing the parasitism rate with higher connectivity [38]. Similarly, the complexity of the searching environment influenced the persistence of *T. nubilale* for host egg masses. Parasitism rates were 2.9 times higher on simpler surfaces than on complex surfaces, and searching time was 1.2 times higher on simple surfaces [39]. Phytochemical cues also affect parasitoid behavior. Numerous studies have found that differences in chemicals present in herbivore frass and volatile chemicals released by plants significantly influenced searching behavior and plant preferences in *Diadegma* spp. [40,41,42,43]. Pugh et al. [15] found that the parasitoid *Cotesia vestalis* exhibited higher rates of parasitism on DBM larvae on VPW than cabbage in both laboratory and field studies. Here, we found some parasitoid-exposed DBM larvae on the wall of the VPW enclosures but not on the wall of the cabbage enclosures, suggesting that the host-searching intensity of *D. insulare* may have been greater on VPW and hence induced a greater avoidance or escape response in DBM. Parasitoid behavior on VPW and cabbage likely stems from differences in both plant structures and chemical compositions [44,45,46].

The host-searching behavior of parasitoids is determined by a combination of innate decision making with no previous host experience and subsequent oviposition experience [47]. Parasitoids used in our study were not exposed to DBM larvae or plants before being introduced to the enclosures though they were reared in cabbage-fed DBM larvae. It is possible that larval experience and/or novelty of VPW could have influenced host searching behaviors. Host density, which affects emission of herbivore-induced plant volatiles and amounts of host frass, can also influence parasitoid host-searching behavior. This has been demonstrated with DBM parasitoids [12,48]. Although we placed equal numbers of DBM larvae in the enclosures, plant structure may have influenced the perception of host density by the parasitoids. For example, we observed DBM frass on cabbage falling mostly into the soil, whereas frass accumulated on the dense foliage of VPW. Thus, *D. insulare* may have spent more time on host searching and consequently harassed more DBM larvae more frequently in the VPW enclosure than in the cabbage enclosure.

Overall, our study demonstrated that DBM larvae suffer physiological costs when threatened by parasitoids. Moreover, we found some evidence to suggest that the severity of the enemy-risk effects is influenced by the DBM host plants. The fact that exposure to parasitoids in the larval stage carries over to influence adult size, which is often correlated with fecundity, suggests that enemy-risk effects may impact DBM population dynamics. Future studies should focus on larger-scale impacts of enemy-risk effects on DBM, including increasing temporal scales over multiple generations and spatial scales in agricultural fields [2,5]. Intercropping with plants that maximize enemy-risk effects on DBM could potentially enhance biological control efforts.

## Data Availability

The data presented in this study are available on request from the corresponding author.
